# Modelling pH-Optimized Degradation of Microgel-Functionalized Polyesters

**DOI:** 10.1155/2016/8125416

**Published:** 2016-08-18

**Authors:** Lisa Bürgermeister, Marcus Hermann, Katalin Fehér, Catalina Molano Lopez, Andrij Pich, Julian Hannen, Felix Vogt, Wolfgang Schulz

**Affiliations:** ^1^Fraunhofer Institute for Laser Technology, Steinbachstrasse 15, 52074 Aachen, Germany; ^2^Nonlinear Dynamics of Laser Processing, RWTH Aachen University, Steinbachstrasse 15, 52074 Aachen, Germany; ^3^Institute of Textile Technology, RWTH Aachen University, Otto-Blumenthal-Strasse 1, 52074 Aachen, Germany; ^4^Functional and Interactive Polymers, DWI, RWTH Aachen University, Forckenbeckstrasse 50, 52074 Aachen, Germany; ^5^Innovation, Strategy and Organization Group, RWTH Aachen University, Kackertstrasse 7, 52072 Aachen, Germany; ^6^Department of Cardiology, RWTH Aachen University, Pauwelstrasse 30, 52074 Aachen, Germany

## Abstract

We establish a novel mathematical model to describe and analyze pH levels in the vicinity of poly(*N*-vinylcaprolactam-co-acetoacetoxyethyl methacrylate-co-*N*-vinylimidazole) (VCL/AAEM/VIm) microgel-functionalized polymers during biodegradation. Biodegradable polymers, especially aliphatic polyesters (polylactide/polyglycolide/polycaprolactone homo- and copolymers), have a large range of medical applications including delivery systems, scaffolds, or stents for the treatment of cardiovascular diseases. Most of those applications are limited by the inherent drop of pH level during the degradation process. The combination of polymers with VCL/AAEM/VIm-microgels, which aims at stabilizing pH levels, is innovative and requires new mathematical models for the prediction of pH level evaluation. The mathematical model consists of a diffusion-reaction PDE system for the degradation including reaction rate equations and diffusion of acidic degradation products into the vicinity. A system of algebraic equations is coupled to the degradation model in order to describe the buffering action of the microgel. The model is validated against the experimental pH-monitored biodegradation of microgel-functionalized polymer foils and is available for the design of microgel-functionalized polymer components.

## 1. Introduction

Cardiovascular diseases are the number one cause of death worldwide, globally claiming 17 million lives each year and accounting for 29% of all deaths [1]. Through the introduction of minimally invasive procedures like percutaneous coronary intervention (PCI) and the use of intravascular stents, the treatment of many cardiovascular diseases such as coronary artery disease, the obstruction of a coronary artery due to development of atherosclerosis, has been revolutionized [2, 3].

Nevertheless, efficacy and safety of available stents are limited in part as suitable autologous tissue engineered stents are lacking [4]. Mostly, nondegradable synthetic materials are substituted to repair the injured cardiovascular tissue. Meant to prevail at the implantation site, these foreign and therefore inherently thrombogenic materials can be associated with several risks, including calcification and acute or late stent thrombosis, which require prevention by antiplatelet therapy [5].

In contrast, tissue engineered bioresorbable stents provide temporary scaffolding for the formation of autologous tissue with the capacity to regenerate and grow. After the degradation process of the stent, only the restored vessel remains which might reduce the risk of late stent thrombosis.

The most frequently used resorbable polymers for biomedical implants including bioresorbable stents are the aliphatic polyesters polylactic acid (PLA) and polyglycolic acid (PGA) and their copolymers. The mechanism of polyester degradation has been well investigated. Polymer chains are degraded by hydrolytic scission of ester linkages in the polymer backbone and thereby create carboxylic end groups. These acidic carboxyl end groups diffuse into the vicinity of the polymer and decrease the pH level there [6–10].

This pH drop is one of the main drawbacks of using aliphatic polyesters for implants as a low pH may cause tissue reactions like inflammation [11–13].

Still, biodegradable stents have marked potential long-term advantages [4, 14]. In order to overcome the disadvantage of acidic pH levels during degradation of biodegradable stents, we investigate the fabrication of polylactic acid (PLA) fibers for cardiovascular stents with pH-optimized degradation behavior using VCL/AAEM/VIm (8 mol%)-microgels as insoluble buffers [15].

Microgels are defined as hydrogel particles with a dimension from 10 nanometers up to the micrometer range. Hydrogels in general are novel components that arouse special interest in the biomedical field and nanotechnology [16, 17].

Depending on the molecular building units and the synthesis procedure, it is possible to synthesize colloidal hydrogels with controlled particle size. Those can be defined as polymer networks with smart properties such as water-uptake capacity, degree of swelling, and responsiveness to external stimuli. Furthermore, these nanoparticles show high colloidal stability, a well-defined structure, and a high surface area. Through variation of the monomer type and/or introduction of comonomers, responsiveness to temperature, pH, magnetic field, or light intensity can be tailored. This property, in combination with the swelling features, makes the utilization of microgels as biocompatible materials for pharmaceutical applications possible [16, 18–23].

Monomers like N-isopropylacrylamide (NIPAAm) and N-vinylcaprolactam (VCL) have been used for the synthesis of thermoresponsive microgels because they can form water-soluble polymers with lower critical solution temperature (LCST) [18, 20, 24, 25]. Other interesting components such as N-vinylimidazole (VIm) can be used to achieve pH-sensitivity due to its protonation/deprotonation process at different pH levels. Pich et al. [26] have successfully synthesized VCL/VIm-based microgels, which show both temperature and pH-responsiveness. In this study, the swelling could be controlled by the VIm content in the microgel.

However, cardiovascular stents require for the degradation period to last over months, making their experimental research as well as their design and optimization rather time-intensive. This study aims at developing a mathematical model able to predict the pH level resulting from polymer degradation and taking into account the buffering action of eventually incorporated microgels. This mathematical model will be validated against the experimental pH-monitored biodegradation of microgel-functionalized polymer foils and will be available for the design of microgel-functionalized polymer stents and other components.

Computational methods to model the degradation of biodegradable polymers have been proposed by a quantity of research teams. Siparsky et al. [27] described the kinetics of hydrolysis reaction by rate equations and their analytic solutions. Han [28] used Monte Carlo methods to predict the degradation of PLA, PGA, and some copolymers. He also investigated the influence of chemical buffers on the degradation process and the pH level. Wang et al. [29] presented a reduced model for polymer degradation using two rate equations for the concentrations of carboxylic end groups and ester bonds in the PLA phase. Pan et al. [30] included a model for chemical buffers in such a reduced model for biodegradation. Moreover, rate equations for the concentrations of different molecules sizes, which the polymer undergoes in its degradation process, were built up and analyzed by Lazzari et al. [31]. The buffering action of the poly(N-vinylimidazole) hydrogel was modelled and analyzed by Horta et al. [32, 33]. To the best of the authors' knowledge, so far no published model for the biodegradation of polymers buffered with VCL/AAEM/VIm-microgels exists.

Investigating microgel-functionalized resorbable polymers with pH-regulatory potential, a recent study of Fehér et al. [15] employs a publication-based keyword search strategy to investigate the existing knowledge base underpinning the topic. The authors find that only a marginal number of studies is dedicated to investigate the pH-regulative potential of microgel containing degradable polymers. This clearly demonstrates an underinvestigated topic. Given the potential scope of application, ranging from drug-coated, pH-regulating degradable textiles to stents, this paper is aligned to the apparent research gap.

## 2. Materials and Methods 

### 2.1. VCL/AAEM/VIm (8 mol%)-Microgel

The synthesis of VCL/AAEM/VIm (8%)-microgel is well described in Fehér et al. [15]. The description is not repeated here.

### 2.2. Polymer VCL/AAEM/VIm (8 mol%)-Microgel Foils

Poly(L-lactide-co-glycolid) PLG 8523 by Purac and the microgel are weighed separately. Both samples are then filled up with a solvent in a way that the mass fraction of polymer *w*_Pol_ and microgel *w*_MG_ amount to 0.15, respectively. The sealed samples are stirred at 250 rpm for 24 hours in a magnetic stirrer. The resulting microgel and polymer solutions are mixed thoroughly to ensure a homogeneous solution. The solutions are evenly spread on a petri dish and thereby infused to a foil. After 48 hours within the distractor hood, the solvent is fully evaporated, allowing for the foil to be peeled from the glass.

### 2.3. Degradation Experiment

The degradation experiments are carried out according to ISO 13781 [34]. Each sample contains 0.1 g of the produced foil and is supplemented with distilled water (specific conductivity < 0.1 *μ*S/cm at 25°C) at the ratio of 1 : 100. The samples are prepared in 20 mL glass containers and sealed during degradation periods. The containers are only opened for pH measurement.

Throughout the degradation process, the samples are stored in an oven at 37°C. pH measurement is conducted daily within the first 20 days followed by weekly readings for a total time period of at least 30 days. To determine the pH level, the samples are withdrawn from the oven. Due to the high temperature sensitivity of the pH level, the samples are cooled down to ambient temperature (20°C) prior to the measurement. Each measurement is repeated twice and the three results are averaged. To increase the experiment's significance, each sample is prepared twice and the average is calculated from the results. To ensure a homogeneous distribution of hydron H^+^ during measuring, the samples are swiveled prior to the measurement. The pH-meter “SevenEasy*™* S20” with an uncertainty of ±0.01 by Mettler Toledo, Gießen, Germany, is used. The electrode of the pH-meter is calibrated beforehand using buffer solutions with pH levels of 4.01, 7.00, and 9.21.

### 2.4. Physical Model Reduction and Mathematical Modelling

In order to analyze the pH level in a vicinity of a degrading polyester component, we predict the concentration of hydron [H^+^] resulting from dissociation of carboxyl groups of the degrading polymer. As the dissociation of the carboxyl groups COOH to COO^−^ and hydron H^+^ in aqueous solution takes place on a very short timescale, we make use of the equilibrium constant *K*_COOH_ = [COO^−^][H^+^]/[COOH] for the dissociation reaction and insert it into the balance of mass and charge to calculate the concentration of hydron [H^+^]:

(1) $$\begin{array}{c} \left[\;H^{+}\;\right]=\frac{K_{w}}{\left[H^{+}\right]}+\frac{K_{COOH}\left[M\right]}{\left[H^{+}\right]+K_{COOH}} \end{array}$$

with the equilibrium constant for the dissociation of H_2_O to OH^−^ and H^+^: *K*_w_ = [H^+^][OH^−^]. The brackets [X] mark the concentration of the corresponding species X. We used the fact that the concentration of oligomers [M] corresponds to the concentration of carboxyl groups [COOH]. The concentration of H_2_O is not part of the equation as we assume it to be constant because the water penetration into the polymer is on a very short timescale.

The concentration of carboxyl groups increases with the progressing scission of polymer chains by hydrolysis reaction and is thus time dependent. Hydrolysis of polyesters proceeds partly as spontaneous reaction and partly catalyzed by hydron H^+^. The reaction is called autocatalytic as the reaction products hydroxyl alcohol and carboxylic acid end groups—also referred to as oligomers [28]—dissociate and acidize the environment and as a result accelerate the rate of hydrolysis [27]. The hydrolysis reaction is explained in detail elsewhere [35].

We implement a phenomenological model for the degradation of biodegradable polymers initially introduced by Wang et al. [29]. As a result, we predict the evolution in time of oligomer concentration [M]_init_ after degradation and before buffering as well as the evolution in time of concentration of ester bonds [E] inside the calculation area by two rate equations:

(2) dEdt=−kE−kcatEH+,

(3) dMinitdt=−dEdt

with the rate coefficients *k* and *k* ^cat^ for spontaneous and catalytic scission of ester bonds inside the polymer, respectively. Using the equilibrium constant *K*_COOH_, we transform (2) to

(4) $$\begin{array}{c} \frac{d\left[E\right]}{dt}=-k\left[\;E\;\right]-k^{cat}\sqrt{K_{COOH}}\left[\;E\;\right]\sqrt{\left[M\right]}. \end{array}$$

[M] refers to the concentration of oligomers available for the autocatalytic scission.

After dissociation of oligomers, the hydrons are buffered by the VIm groups of the microgel. Microgel and polymer form a two-phase system. The hydrons diffuse inside the microgel and are bound to the VIm. The dissociation products COO^−^ bound to the oligomers will also diffuse inside the microgel and remain there unbound due to electrostatic forces. The diffusion processes will continue until the thermodynamic equilibrium between the two phases is reached. Using the assumption that the buffer reaction is fast compared to the degradation processes, we use the equilibrium constant of the buffer reaction *K*_P_ = [P][H^+^]/[PH^+^] with the concentration of free buffer molecules [P] and occupied buffer molecules [PH^+^], respectively, and find a system of algebraic equations describing the buffering action of the microgel. The derivation is explained in detail for poly(N-vinylimidazole) by Horta and Piérola [33]:

(5) COO−gel=H+gel3+H+gel2KP+P0−H+gel·Kw−KP·KwH+gel·H+gel+KP,COO−gel=−H+−KwH+·VbathVgel+H+init−KwH+init·VinitVgel,H+=H+gelCOO−gel+Kw,

where [X]_gel_ represents the concentration of the species X in the microgel phase, [X]_init_ represents the concentration in the polymer before the buffering reaction, *V*_init/gel/bath_ are the volumes occupied by the polymer without the gel, the volume of the microgel, and the volume of the polymer including the microgels, respectively, and [P]_0_ is the initial concentration of buffer molecules in the system. Using (1), we calculate from [H^+^] the concentration of oligomers [M]_bath_ after the buffering action of the microgel and complete the ODE system ((2) + (3)) by a rate equation for the concentration of oligomers [M] after buffering which allows for diffusion of the oligomers:

(6) $$\begin{array}{c} \frac{d\left[M\right]}{dt}=\frac{{\left[M\right]}_{bath}}{{\left[M\right]}_{init}}\left(\;-\frac{d\left[E\right]}{dt}\;\right)+D_{0}\Delta \left[\;M\;\right] \end{array}$$

with *D*_0_ being a constant diffusivity of oligomers.

The calculation area and initial and boundary conditions are chosen appropriate for the degradation experiment described above. As a consequence of the typical extensions of a polymer foil, diffusion will be dominant in one dimension. Thus, we set up a 1D diffusion model and use *x* as the coordinate in space. The calculation area consists of two components: the (microgel containing) polymer with the thickness *d*_p_ and the surrounding water with the thickness *d*_w_. At time *t* = 0, ester bonds have the initial concentration [E]_0_ in the polymer foil (0 ≤ *x* ≤ *d*_p_) and 0 in the water volume (*x* > *d*_p_) and the initial concentrations of oligomers [M]_init_ and [M] are 0 everywhere:

(7) Et=00≤x≤dp=E0,Et=0x>dp=0,Mt=0=Minitt=0=0.

The boundaries at *x* = 0 and *x* = *d*_w_ are isolated and do not allow for diffusion of oligomers. At the boundary *x* = *d*_p_ continuity of oligomers is required:

(8) dMdxx=0=dMdxx=dw=0,Mx=dp−=Mx=dp+.

**Figure 1:**
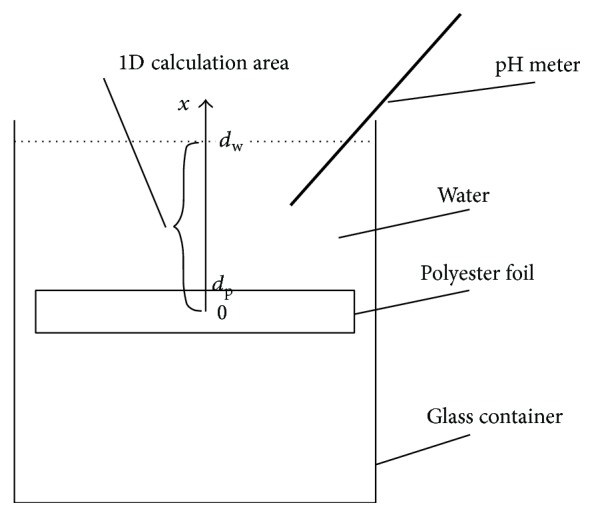
Schematic representation of the 1D simulation area, where *x* depicts the coordinate in space and *d*_p_ and *d*_w_ are the positions of the boundary of polymer foil and water layer, respectively.

A schematic representation of experimental set-up and the calculation area is depicted in Figure 1.

The diffusivity of oligomers *D*_0,p_ and *D*_0,w_ is considered to be constant in the polymer and the water layer, respectively. In order to simulate the swiveling of the samples in the experimental procedure, we increase the diffusion constant *D*_0,w_ in (6) by a factor of 10^3^ for a very short period on every simulated measurement event.

The parameters which have been used to carry out the simulation experiments, discussed in the following chapter, are listed in Table 1.

## 3. Results and Discussion

### 3.1. Buffering Action of VCL/AAEM/VIm-Microgels

**Figure 2:**
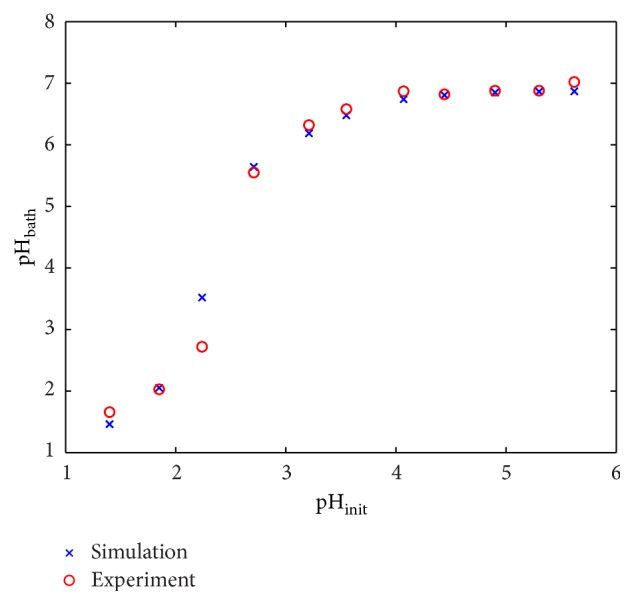
pH level (pH_bath_) of different initial concentrations of hydrochloric acid (pH_init_) after buffering with 0.1 g microgel.

As the buffering model (5) was originally applied to a poly(N-vinylimidazole) gel [33], we investigate its appropriateness for the VCL/AAEM/VIm-microgels in a preliminary study. Therefore, we adjust hydrochloric acid to different initial pH levels pH_init_, add 0.1 g of VCL/AAEM/VIm-microgel, and measure the pH level of the common bath pH_bath_. We calculate the resulting pH levels pH_bath_ using (5) and the same initial pH levels as in the experiment. The simulation result in comparison to the experiment is plotted in Figure 2. We consider the unknown equilibrium constant for the buffer reaction *K*_P_ and the amount of VIm molecules per microgel unit *σ* as fitting parameters and use the withdrawn values of *K*_P_ = 1 · 10^−10^  mol/L and *σ* = 1,522 · 10^20^ 1/g for further simulation. The preliminary study shows a threshold behavior for the buffering effect. Initial pH levels above 4 (pH_init_ ≥ 4) are buffered by VCL/AAEM/VIm-microgel to a neutral pH level. For initial pH levels below this value, the buffering action of VCL/AAEM/VIm-microgel in the used concentration is not sufficient to receive a neutral pH level.

**Figure 3:**
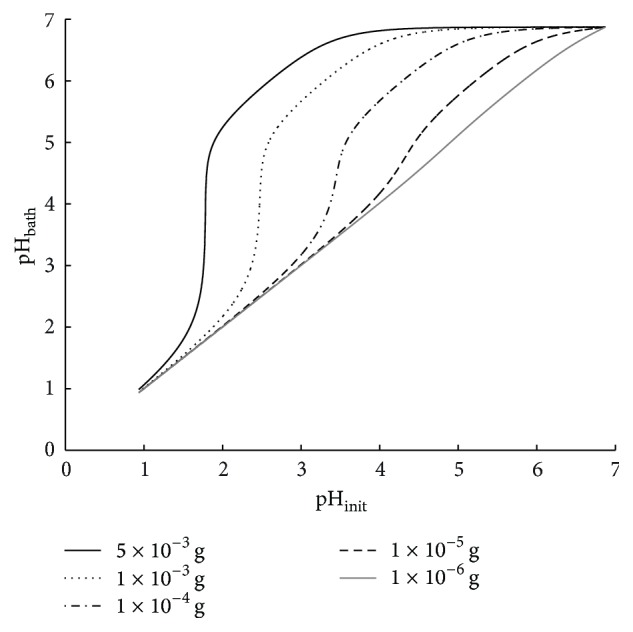
Predicted pH-value (pH_bath_) after buffering different initial pH levels (pH_init_) with different constant microgel amount.

Furthermore, we analyze the amount of VCL/AAEM/VIm-microgel necessary for successful buffering action to a neutral pH level. The result is shown in Figure 3. Initial pH levels of 3.75 and higher can be buffered to neutral level by a total amount of 0.005 g microgel (solid black line in Figure 3). This corresponds to a microgel mass fraction *w*_MG_ of 0.05 in the polymer foils (for preparation, see above). A threshold below which very strong decay of pH level arises is seen at pH_init_ = 1.78 for the 0.005 g VCL/AAEM/VIm-microgel. Lower VCL/AAEM/VIm-microgel amounts (non-solid and gray lines in Figure 3) lead to higher threshold pH levels. Thus, the curves in Figure 3 are shifted to the right with decreasing VCL/AAEM/VIm-microgel amount. The threshold behavior is less distinct for lower microgel amounts. Hence, the curves in Figure 3 are also flattened with decreasing VCL/AAEM/VIm-microgel amount. Nearly no buffering action is predicted for a mass of 1 · 10^−6^ g VCL/AAEM/VIm-microgel.

This analysis is valid for the ideal case when considering a homogenous VCL/AAEM/VIm-microgel distribution inside the polymer, where diffusion of oligomers to reach the VIm-molecules is not necessary and no barriers, for example, electrostatic reasons, occur.

### 3.2. Degradation Studies

**Figure 4:**
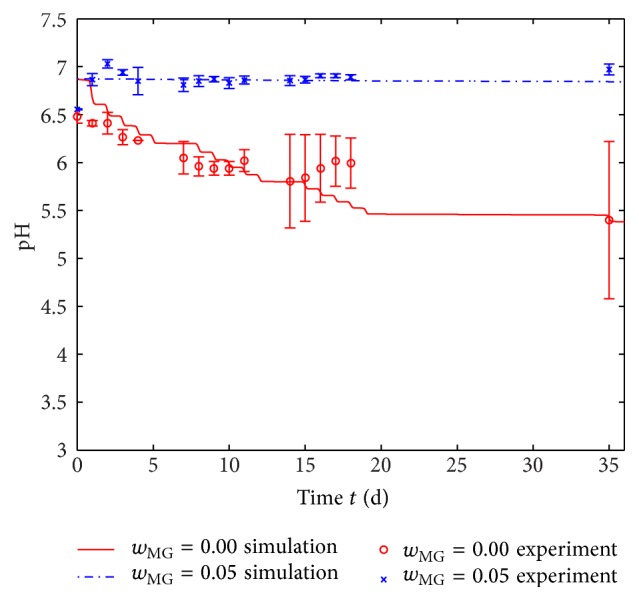
pH level over time during degradation experiment of polymer foils with a VCL/AAEM/VIm-microgel mass fraction *w*_MG_ of 0.00 and 0.05, respectively, in comparison to the simulation (for evaluation in water component, see Figure 1). The model parameters are given in Table 1.

The experimental results from the degradation studies of polymer foils with VCLVCL/AAEM/VIm-microgel mass fractions of *w*_MG_ = 0.00 and *w*_MG_ = 0.05, respectively, are depicted in Figure 4 via the mean and the standard deviation of the two measurement series (see above). Without microgel, a drop in pH level arises as expected leading to pH = 5.4 after 35 days. As the pH level is measured in the water component of the experimental set-up (*d*_p_ ≤ *x* ≤ *d*_w_ in Figure 1), the pH level in the core of the polymer foil (*x* = 0) will be lower. This lowest pH level is relevant to choosing the appropriate VCL/AAEM/VIm-microgel content.

We use the experimental results of polymer foils with a microgel mass fraction of *w*_MG_ = 0.00 to determine the rate constants in the degradation model (2) in order to find a good agreement between simulation and experimental measurements. The simulation result as average pH level in the water component (*d*_p_ ≤ *x* ≤ *d*_w_ in Figure 1) is plotted as a solid line in Figure 4. The influence of the simulated swiveling process can be seen clearly as steps in the pH level curve. The obtained degradation rates as all other model parameters are listed in Table 1.

**Figure 5:**
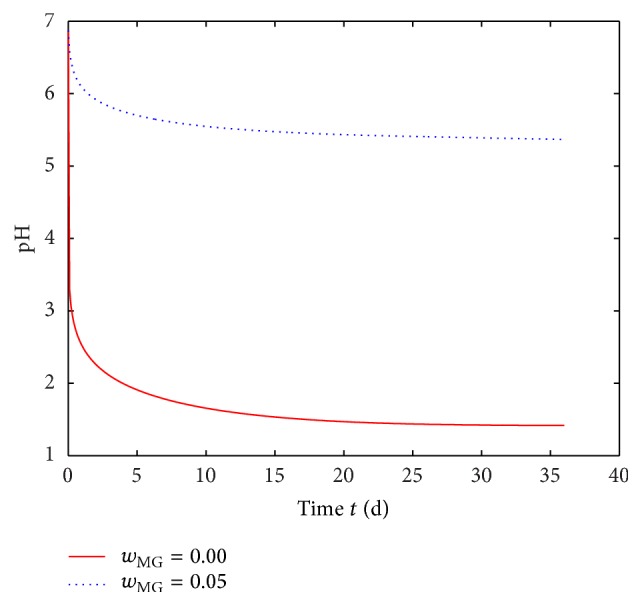
Simulated pH level over time for polymer foils with a VCL/AAEM/VIm -microgel mass fraction of *w*_MG_ = 0.00 and *w*_MG_ = 0.05, respectively. The pH level was evaluated at the center of the polymer foil (*x* = 0 in Figure 1). The model parameters are given in Table 1.

**Figure 6:**
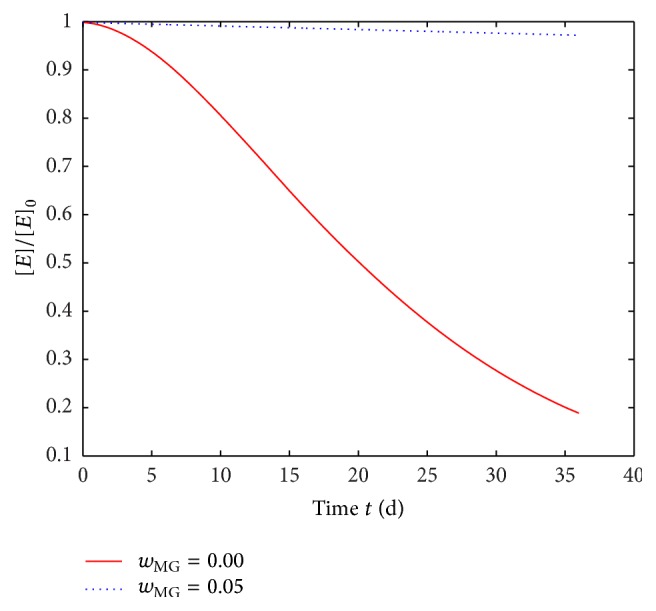
Simulated normalized ester bond concentration [E]/[E]_0_  over time for polymer foils with a VCL/AAEM/VIm-microgel mass fraction of *w*_MG_ = 0.00 and *w*_MG_ = 0.05, respectively. The concentration was evaluated as an average of the polymer foil (0 < *x* < *d*_p_ in Figure 1). The model parameters are given in Table 1.

The pH level in the center of the polymer foil (*x* = 0 in Figure 1) is evaluated and plotted over time in Figure 5. The pH level drops to 1.42 after 35 days using a mass fraction of *w*_MG_ = 0.00 (solid line). Applying a VCL/AAEM/VIm-microgel buffer with a mass fraction of *w*_MG_ = 0.05 to the resulting solution (pH_init_ = 1.42) will not be sufficient to buffer the pH to a neutral level but only to a pH level of approximately pH_bath_ = 2 according to Figure 3. Nevertheless, in the center of the foil after 35 days only a small decrease in pH level (~5.37) is predicted for a microgel mass fraction of *w*_MG_ = 0.05 (dotted line in Figure 5). This discrepancy results from the deceleration of the degradation process through VCL/AAEM/VIm-microgel. The deceleration can be explained via the reduction of the autocatalyzed degradation. The normalized concentration of ester bonds in the polymer component over time is depicted in Figure 6. It illustrates the reduction of overall degradation rate by incorporation of VCL/AAEM/VIm-microgel. Thus, a VCL/AAEM/VIm-microgel mass fraction of *w*_MG_ = 0.05 will possibly be sufficient to buffer the pH to a neutral level. Figure 4 confirms this expectation in theory and experiment. The predicted decrease of pH level in the water component is smaller than in the center of the foil and is in good accordance with the measurements.

**Figure 7:**
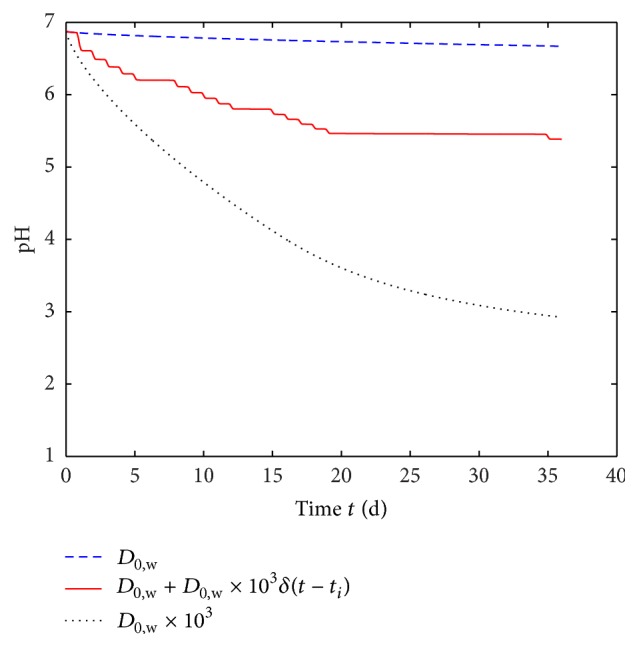
Simulated pH level over time for polymer foils without any VCL/AAEM/VIm-microgel content. The diffusivity was changed from a low value (*D*_0,w_) to a higher value (*D*_0,w_ · 10^3^) to a low value with several peaks *i*  (*D*_0,w_ + *D*_0,w_ · 10^3^ *δ*(*t* − *t*_*i*_) with the Kronecker delta *δ*). The pH level was evaluated as an average of the water phase (*d*_p_ < *x* < *d*_w_ in Figure 1). The model parameters are given in Table 1.

In order to briefly demonstrate the influence of the experimental procedure on the development of pH level, three different swiveling procedures are simulated and the resulting average pH levels in the water phase are plotted in Figure 7 (no swiveling: dashed line, swiveling at discrete measurement events: solid line, and continuous swiveling: dotted line). The strong deviation in simulated pH levels shows the importance of standardized experimental procedures.

### 3.3. Limitations of the Study

The rate equation system for degradation of polymers ((1)–(3) and (6)) is a very reduced model. Any difference between scission of ester bonds at molecule ends and that of bonds in the middle of the polymer molecules is neglected as well as the effects of crystallinity, of diffusivity varying in time, and others. Nevertheless, a very similar model is already considered very useful for the prediction of size and shape effects on biodegradation [29]. One has to perform reference experiments without incorporation of VCL/AAEM/VIm-microgels to determine the rate coefficients of the presented degradation model. With these references our model can be used for investigating the pH levels in the vicinity of polymer components and for analyzing buffering behavior. The reference experiments will have different results using different polymers and therefore have to be performed for every material that is investigated.

The simulated model task consisting of a 1D calculation area is a simplified task and deviations from experimental measurements are expectable. It is adequate for a first benchmark of the model with experiments and appropriate for model development as very short computation time results from the simple task.

Model refinements and extensions are necessary in order to investigate the influence of microgel distribution inside the polymer and buffering reaction kinetics. For this purpose, new specific experiments have to be conducted.

We study the degradation behavior of polymer foils in a preclinical set-up according to ISO 13781. Any biological influences like enzymes, vascularization, buffering in body fluids, or patient individual factors cannot be investigated in degradation experiments of this kind. As can be seen from simulation results (Figure 7), conditions (e.g., flow of liquid medium) during degradation experiments have an important influence on the degradation results. Thus, the authors suggest building up a more complex test bench for degradation experiments simulating blood flow amongst others before beginning with clinical studies.

## 4. Conclusions

VCL/AAEM/VIm-microgel-functionalized fibers with pH-optimized degradation behavior are a promising approach for a wide range of medical applications. In particular, the treatment of many cardiovascular diseases such as coronary artery disease will benefit from biodegradable material for stents without the drawback of acidic pH levels as a consequence of degradation.

Within this study, a mathematical model is presented, which can deal as an important tool to design components with pH-optimized degradation behavior. Iterative design of suitable degradation behavior based on mathematical modelling is shown to exploit the potentials of the medical application and will save engineering costs and time for degradable polymer devices and can contribute to reducing animal studies.

The mathematical model consists of a reduced degradation model based on a rate equation system including diffusion of acidic degradation products into the vicinity of the component. This degradation model is coupled with a set of algebraic equations modelling the buffering action of VCL/AAEM/VIm-microgel. Both models (rate equations for degradation and algebraic buffering model) are evaluated separately in comparison to experimental measurements of pH level and show good agreement with them.

The pH level during the degradation of polymer foils is monitored during 35 days and serves as reference to fit the rate constants of the mathematical degradation model. Additionally, initial pH levels are buffered with a constant amount of VCL/AAEM/VIm-microgel to calibrate the model for the buffer reaction.

A VCL/AAEM/VIm-microgel mass fraction of *w*_MG_ = 0.05 is found to deliver a sufficient buffering action within at least 35 days of degradation of polymer foils. Simulation as well as experimental studies confirmed this buffering effect.

## Figures and Tables

**Table 1:** Parameters used for simulation.

Symbol	Value	Unit	Description	Source
*K*_COOH_	10^−3,87^	mol/L	Equilibrium constant of dissociation reaction of COOH at 37°C	[28]
*K*_*w*_	1.8 · 10^−14^	(mol/L)^2^	Equilibrium constant of dissociation reaction of H_2_O at 37°C	[36]
*k*	0.005	1/week	Rate constant of spontaneous ester bond scission	Fit to experiments
$k^{cat}\cdot \sqrt{K_{COOH}}$	0.5 · 10^−3^	$\sqrt{m^{3}/mol}/week$	Rate constant for autocatalyzed ester bond scission	Fit to experiments
*K*_P_	1 · 10^−10^	mol/L	Equilibrium constant for buffer reaction at 20°C	Fit to experiments
[P]_0_	0.3487	mol/L	Initial buffer concentration	Fit to experiments
*V*_gel_/*V*_init_	9.42 · 10^−6^	—	Fraction of volumes occupied by gel and polymer	Derived from experimental procedure
*V*_bath_/*V*_init_	1 − *V*_gel_/*V*_init_	—	Fraction of volumes occupied by polymer + gel and polymer	—
*D*_0,p_	2 · 10^−9^	m^2^/week	Diffusion constant of oligomer in the polymer	[28]
*D*_0,w_	2 · 10^−8^	m^2^/week	Diffusion constant of oligomer in water	[37]
*d*_p_	0.15	mm	Thickness of polymer foil	Derived from experimental procedure
*d*_w_	19.6	mm	Thickness of water column above polymer foil	Derived from experimental procedure
